# Unique Behavioral Characteristics and microRNA Signatures in a Drug Resistant Epilepsy Model

**DOI:** 10.1371/journal.pone.0085617

**Published:** 2014-01-15

**Authors:** Jangsup Moon, Soon-Tae Lee, Jiye Choi, Keun-Hwa Jung, Hyunwoo Yang, Arshi Khalid, Jeong-Min Kim, Kyung-Il Park, Jung-Won Shin, Jae-Jun Ban, Gwan-Su Yi, Sang Kun Lee, Daejong Jeon, Kon Chu

**Affiliations:** 1 Laboratory for Neurotherapeutics, Department of Neurology, Comprehensive Epilepsy Center, Biomedical Research Institute, Seoul National University Hospital, Seoul National University College of Medicine, Seoul, Republic of Korea; 2 Department of Bio and Brain Engineering, Korea Advanced Institute of Science and Technology (KAIST), Daejeon, Republic of Korea; 3 Department of Neurology, Chung-Ang University Hospital, Chung-Ang University College of Medicine, Seoul, Republic of Korea; 4 Department of Neurology, Seoul Paik Hospital, Inje University College of Medicine, Seoul, Republic of Korea; Queen's University Belfast, United Kingdom

## Abstract

**Background:**

Pharmacoresistance is a major issue in the treatment of epilepsy. However, the mechanism underlying pharmacoresistance to antiepileptic drugs (AEDs) is still unclear, and few animal models have been established for studying drug resistant epilepsy (DRE). In our study, spontaneous recurrent seizures (SRSs) were investigated by video-EEG monitoring during the entire procedure.

**Methods/Principal Findings:**

In the mouse pilocarpine-induced epilepsy model, we administered levetiracetam (LEV) and valproate (VPA) in sequence. AED-responsive and AED-resistant mice were naturally selected after 7-day treatment of LEV and VPA. Behavioral tests (open field, object exploration, elevated plus maze, and light-dark transition test) and a microRNA microarray test were performed. Among the 37 epileptic mice with SRS, 23 showed significantly fewer SRSs during administration of LEV (n = 16, LEV sensitive (LS) group) or VPA (n = 7, LEV resistant/VPA sensitive (LRVS) group), while 7 epileptic mice did not show any amelioration with either of the AEDs (n = 7, multidrug resistant (MDR) group). On the behavioral assessment, MDR mice displayed distinctive behaviors in the object exploration and elevated plus maze tests, which were not observed in the LS group. Expression of miRNA was altered in LS and MDR groups, and we identified 4 miRNAs (miR-206, miR-374, miR-468, and miR-142-5p), which were differently modulated in the MDR group versus both control and LS groups.

**Conclusion:**

This is the first study to identify a pharmacoresistant subgroup, resistant to 2 AEDs, in the pilocarpine-induced epilepsy model. We hypothesize that modulation of the identified miRNAs may play a key role in developing pharmacoresistance and behavioral alterations in the MDR group.

## Introduction

Pharmacoresistance is a major issue in the treatment of epilepsy. About 30–40% of patients do not respond to treatment with different antiepileptic drugs (AEDs) [1]–[3]. The International League Against Epilepsy (ILAE) defines drug resistant epilepsy (DRE) as the “failure of adequate trials of two tolerated and appropriately chosen and used AED schedules to achieve sustained seizure freedom.” [4] However, the mechanisms underlying pharmacoresistance are unclear.

Animal models of DRE may help elucidate the underlying mechanisms and develop new treatment strategies for refractory epilepsies. Considering the ILAE definition [4], the term “pharmacoresistance” in animal models can be defined as persistent seizure with poor response to at least two AEDs of empirically efficacious doses [5]. Several animal models of DRE have been developed, which can be categorized into two groups. The first group demonstrates seizures or epilepsy that *per se* are resistant to AEDs [6]–[8]. The second group includes an AED resistant subgroup in an established epilepsy model [9]–[11]. Using these animal models, some mechanisms of pharmacoresistance have been identified [12]. Post-status epilepticus (SE) model of temporal lobe epilepsy (TLE) is the most widely used model of the latter group. However studies demonstrating animals that show poor response to more than 2 AEDs are scarce [13].

Epilepsy is associated with psychiatric comorbidities, including cognitive impairment, depression, and anxiety disorders [14], [15]. The relationship between epilepsy and psychiatric comorbidities is suggested to be bidirectional and it is known that patients with DRE are at higher risk to develop a psychiatric disorder [16], [17]. Similar abnormal behaviors are also observed in animal models of epilepsy [18], [19], so we supposed that DRE animals might show distinctive behaviors.

Epigenetics comprises the study of changes in gene expression or cellular phenotype that are not due to changes in DNA sequence [20]. Epigenetic mechanisms play an important role in the development of various neurological diseases [21]. MicroRNAs (miRNAs) take part in the epigenetic process and can be used as biomarkers of epigenetic changes [22]. We presumed that miRNA related epigenetic mechanism could contribute to the generation of DRE and alteration of the behaviors.

This study illustrates a pharmacoresistant subgroup in the widely used pilocarpine-induced epilepsy model in mice and demonstrates its behavioral and epigenetic characteristics.

## Materials and Methods

### Generation of the epilepsy model

Young male C57BL/6J mice (22–25 g) were used in the experiments. The epilepsy model was generated by a single systemic injection of pilocarpine (330 mg/kg, i.p., Sigma) [23]. Methylscopolamine (1 mg/kg, i.p., Sigma) was administered 30 min before the injection of pilocarpine to minimize the peripheral muscarinic effects. Diazepam (5 mg/kg, i.p.) was given 40 min after the onset of SE to interrupt the prolonged seizures (i.e., SE). The onset of SE was defined as the beginning of continuous tonic-clonic behavioral seizures after several discontinuous convulsive seizures (stage 4–6) (the seizure stages were described in below) [24].

After onset of SE, all animals were fed with a 5% glucose solution for 2 days and with soaked food until they were able to eat normal food pellets. Mice were raised with a 12-h light/dark cycle and *ad libitum* access to food and water. Thirty days after SE, EEG surgery was performed. Sixty days after SE, the animals developed spontaneous recurrent seizures (SRSs) and experiments were performed. Mice only receiving methylscopolamine were designated as the control group. The control group also underwent EEG surgery, in order to minimize the impact of surgery on the test results. All procedures in animal care and handling were approved by the Institutional Animal Care and Use Committee at Seoul National University Hospital and the Korea Advanced Institute of Science and Technology (KAIST).

### *In vivo* electrophysiology and analysis of seizure

EEG recording *in vivo* was performed as described previously [23], [25]. Animals were anesthetized by intraperitoneal injection of 1% ketamine (30 mg/kg) and xylazine hydrochloride (4 mg/kg). The surgery was performed using a stereotaxic apparatus (Kopf Instruments) and EEG recordings were obtained using tungsten electrodes (0.005 inch, 2 MΩ), which were positioned into the right hemisphere at AP −1.8 mm, L 2.1 mm, and DV 0.8–1.0 mm (primary somatosensory cortex) from bregma, with grounding over the cerebellum. For perioperative analgesia, local anesthetic (lidocaine 1%, transdermal) was used.

**Figure 1:**
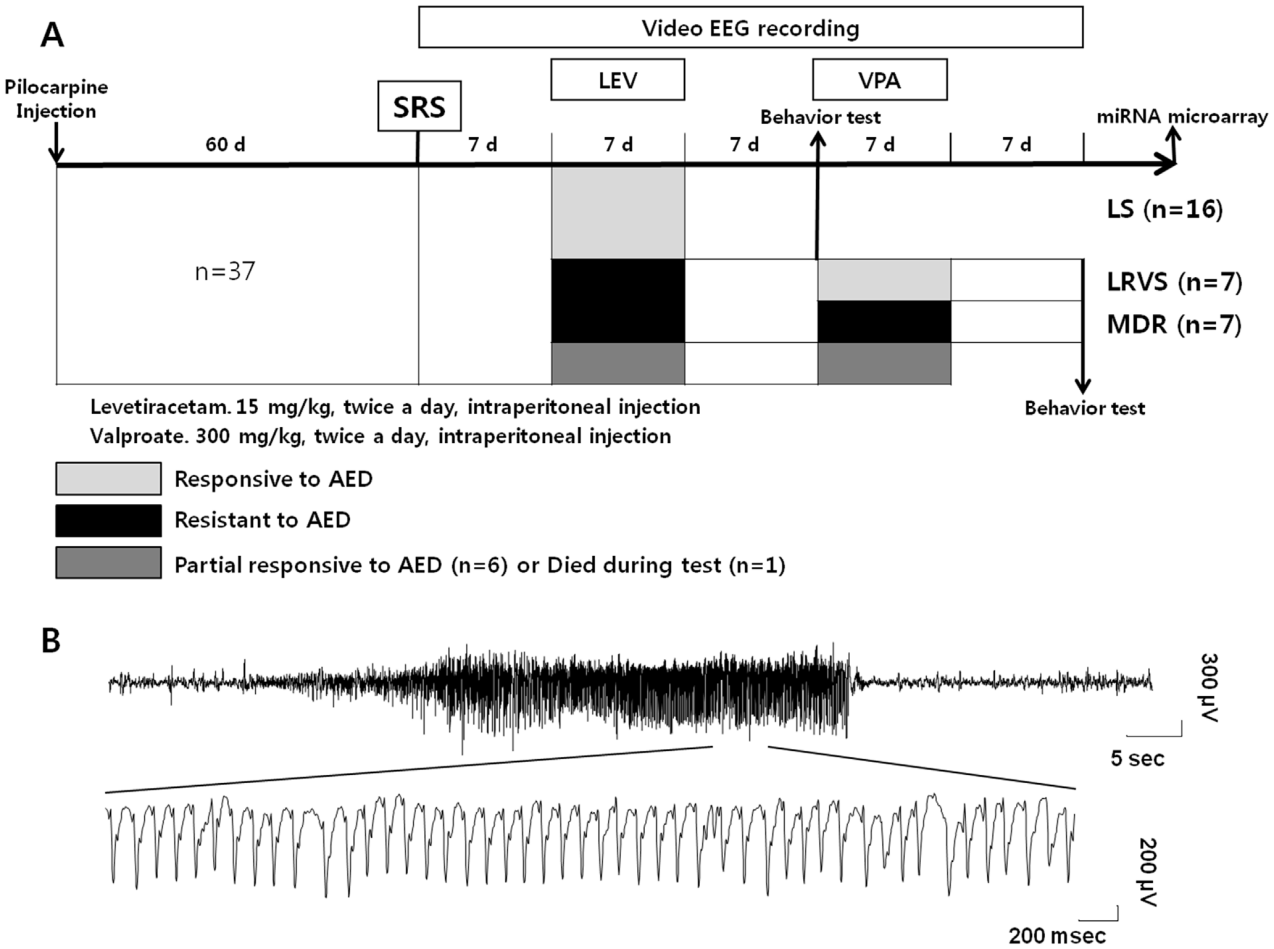
The schematic representation of the study protocol and a representative electroencephalogram data. (**A**) Sixty days after the pilocarpine induction of status epilepticus (SE), continuous video EEG monitoring was started. After 7 days of baseline recording, levetiracetam (LEV) was administered intraperitoneally (15 mg/kg/day) for 7 days. After a washout period of 7 days, valproate (VPA) was given intraperitoneally (30 mg/kg/day) for 7 days. Behavioral tests were performed following a 7-day washout period starting after the last administration of an AED. After all behavioral tests were completed, mice were sacrificed, and a microRNA microarray was performed. Control group is not illustrated because it was only exposed to mehylscopolamine and did not received pilocarpine. (B) Representative electroencephalogram (EEG) traces of SRS. Abbreviations: AED: antiepileptic drug, d: days, SRS: spontaneous recurrent seizure, LS: levetiracetam sensitive, LRVS: levetiracetam resistant/valproate sensitive, MDR: multidrug resistant.

Continuous EEG recordings were combined with video monitoring. From 60 days after SE and until the end of experiments, the video-EEG signals were continuously recorded 24 hr per day (Fig. 1A). Electrical activities were recorded after being amplified (×1200), bandpass-filtered from 0.1 to 70 Hz, and digitized at a 400-Hz sampling rate (AS 40) by a digital EEG system (Comet XL, Astro-Med, Inc., Warwick, RI). Electrographic seizures were defined as the EEG signals showing the changes in the amplitude (>2× background) and frequency of the EEG activity (repetitive spiking with a frequency of 4–12/sec and lasting for at least 10 sec). Behavioral seizures were confirmed by the concurrent video-monitoring according to Racine's scale: [26] stage 1: immobility and rigid posture; stage 2: mouth movements, head nodding, and repetitive movements; stage 3: forelimb clonus; stage 4: severe seizures with rearing and falling; stage 5: severe seizures with loss of posture or jumping; and stage 6: tonic–clonic seizures. Electroclinical SRS was defined as convulsive seizures (stage 4–6) along with epileptic spikes on EEG. The SRS data were averaged and analyzed in 7-day blocks.

### Antiepileptic drugs administration

The baseline SRS frequency was evaluated by EEG recording for 7 days, with intraperitoneal injection of saline twice a day. After that, i.p. levetiracetam (LEV) (15 mg/kg, UCB) was given twice a day for 7 days. After the administration of LEV, every mouse underwent 7 days of washout. During the washout period, only i.p. saline was given twice a day. To the mice that were resistant to LEV, valproate (VPA) (300 mg/kg, Bukwang pharmaceutical) was administered intraperitoneally twice a day for 7 days, followed by a 7-day washout period. During every 7-day block in the monitoring period, EEG was recorded and the alteration in SRS frequency was evaluated (Fig. 1A and B). The doses of AEDs were chosen according to previous studies that demonstrated successful seizure control in animal models [27], [28].

### Evaluation of responsiveness to antiepileptic drugs

We used more strict definition than those used in many AED trials (>50% seizure reduction) for selecting the AED responsive or nonresponsive animals, because we thought comparing the extreme phenotype of each group would reveal the difference better. When the number of SRSs per day during the 7-day AED trial block was reduced to below 25% of the baseline, the mouse was designated to be responsive to that AED. When the number of SRSs per day was over 75% of the baseline, the mouse was presumed to be resistant to that AED. The range between 25 and 75% of the baseline was considered partially responsive.

### Behavioral tests

Open-field, object exploration, elevated plus maze, and light-dark transition tests were used for assessment of anxiety and locomotor activity. Behavioral tests were conducted between 4PM and 8PM at the light intensity of 80 lux, and performed as described in detail previously [29]. The tests were performed in following orders, leaving 7-day intervals between each test: elevated plus maze test, light-dark transition test, and open field test/objection exploration test. The protocols of the tests were applied the same way for all treatment groups. Every mouse underwent video recording during every test and the mice which demonstrated seizure just before or during the tests were excluded from the analysis.

The open-field box was made of white plastic (40×40×40 cm) and the open field was divided into a central field (center, 20 cm×20 cm) and an outer field (periphery). Individual mice were placed in the periphery of the field and the paths of the animals were recorded with a video camera. The total distance travelled for 10 min and the time spent in each field for 5 min were analyzed using EthoVision XT (Noldus). The object exploration test was performed in the same box. Immediately after an open-field test, one object was placed in the center of the box and mice were allowed to explore the object for 5 min.

The elevated plus maze was made of plastic and consisted of two white open arms (25×8 cm), two black enclosed arms (25×8×20 cm), and a central platform (8×8×8 cm) in the form of a cross. The maze was placed 50 cm above the floor. Mice were individually placed in the center with their heads directed toward one of the closed arms. The total time spent in each arm or in the center, and the total number of entries into each arm, were analyzed by video monitoring for 5 min.

The light/dark box (30×45×27 cm) was made of plastic and had a dark compartment (one third of the total area) and a light compartment with a hole in the middle. The light compartment was illuminated at 400 lux. The elapsed time of entry into the light compartment and the amount of time spent in each compartment were measured over a 5-min period by video monitoring.

### miRNA microarray

A miRNA microarray was performed after the behavioral tests were completed. Total RNA was isolated from the individual mouse brains (from a whole brain except cerebellum) with Trizol (Invitrogen, Carlsbad, CA).

Expression profiles for miRNA were examined using an Agilent Mouse miRNA Microarray 8×15K kit according to the manufacturer's protocol (Agilent Technologies, Santa Clara, CA). The scanning and analysis were performed on an Agilent hardware platform, and the obtained data were evaluated using GeneSpring GX software 7.3.1 (Agilent Technologies). Expression changes in miRNAs of more than twofold and a P-value <0.05 were considered significantly different between two groups.

### Statistical analysis

In the animal tests, all data are presented as means ± standard error of mean (SEM), and the Mann-Whitney U test was used for intergroup comparisons. For intergroup comparisons of miRNA expression signature, Student's *t*-test was used. SPSS 21.0 (SPSS Inc, Chicago, Ill) was used for the statistical analyses and a P-value <0.05 was considered significant.

## Results

### Different patterns of antiepileptic drugs response among subgroups of the pilocarpine model

In 45 mice, pilocarpine was injected to induce SE and 8 mice died during the prolonged SE. Among the 37 epileptic mice with SRSs (Fig. 1B), 23 showed substantial reduction in the number of SRSs in response to either LEV or VPA treatment, while 7 were resistant to both AEDs. After treatment with LEV for 7 days, 16 of the 37 epileptic mice showed a significant reduction in SRSs per day during the treatment (*p*<0.001; Table 1). During the 7-day washout, the number of SRSs per day was restored to the pretreatment level (Table 1). After the washout, VPA was given to the mice resistant to LEV. Among them, 7 epileptic mice showed a significant reduction in the number of SRSs per day during VPA treatment (*p*<0.05; Table 1). In 7 epileptic mice, the number of SRSs per day was not reduced in response to either LEV or VPA (Table 1). Six epileptic mice showed a partial response to either LEV or VPA, and were excluded from the analysis (data not shown). One mouse died during the experiment and was excluded.

**Table 1:** Seizure frequency after AED administration in each group.

Group	Baseline	LEV	Washout	VPA
LS (n = 16)	4.29±0.84	0.29±0.12*	4.96±0.7	
LRVS (n = 7)	4.09±1.11	5.41±1.31	5±1.17	0.41±0.35**
MDR (n = 7)	3.57±1.01	6.84±1.64	4.05±0.99	6.01±1.31

All data are presented as mean ± standard error of mean (SEM).

Abbreviations: AED: antiepileptic drug, LEV: levetiracetam, VPA: valproate, LS: levetiracetam sensitive, LRVS: levetiracetam resistant/valproate sensitive, MDR: multidrug resistant.

^() and ()^ represent significant difference between AED trial with its respective baseline (p<0.001 and p<0.05, respectively, Mann-Whitney U test).

The mice that were responsive to initial LEV treatment were designated as the LEV sensitive (LS) group, and the mice responsive to neither LEV nor VPA were labelled as the multidrug resistant (MDR) group. The mice resistant to LEV but responsive to VPA were designated as the LEV resistant/VPA sensitive (LRVS) group.

### Distinctive behavioral characteristics according to the responsiveness to antiepileptic drugs

**Figure 2:**
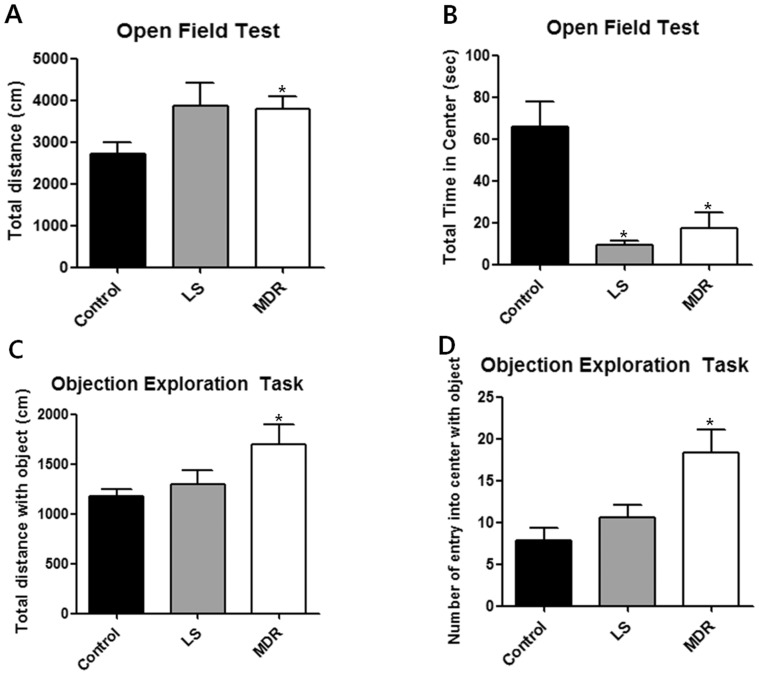
Behavior of mice in each group in the open field and object exploration test. Data are shown as mean ± SEM. (A–B) Open field test (control, n = 6; LS group, n = 11; MDR group, n = 7). (A) illustrates the total distance that the mice moved during the 10 min of the open field test. (B) illustrates the time that mice spent in the aversive center of the open field during 5 min. (C–D) Object exploration test (control, n = 6; LS group, n = 8; MDR group, n = 5). (C) illustrates the total distance that the mice moved during 5 min after one object was placed in the center of the box. (D) illustrates the number of entries into the center during 5 min after one object was placed in the center of the box. (*) represents a significant individual difference when compared to the normal group (p<0.05, Mann-Whitney U test).

We investigated anxiety- and locomotion/exploration-related behaviors in the LS and MDR groups. In the open-field test, total distance moved was significantly increased in the MDR group compared to the control group (*p*<0.05; Fig. 2A). A similar increase of locomotor activity was observed in the LS group, although it was not statistically significant (Fig. 2A). However, both the LS and MDR groups spent less time in the center field than the control group (*p*<0.05; Fig. 2B). There was no significant difference in total time spent in the center between the LS and MDR groups (Fig. 2B). In the object exploration test, the MDR group showed a significant increase in total distance moved (*p*<0.05) and number of entries into the center field with an object (*p*<0.05) compared to the control group. However, there was no significant difference in total distance moved (Fig. 2C) or number of entries into the center field with an object (Fig. 2D) between the control and LS groups.

**Figure 3:**
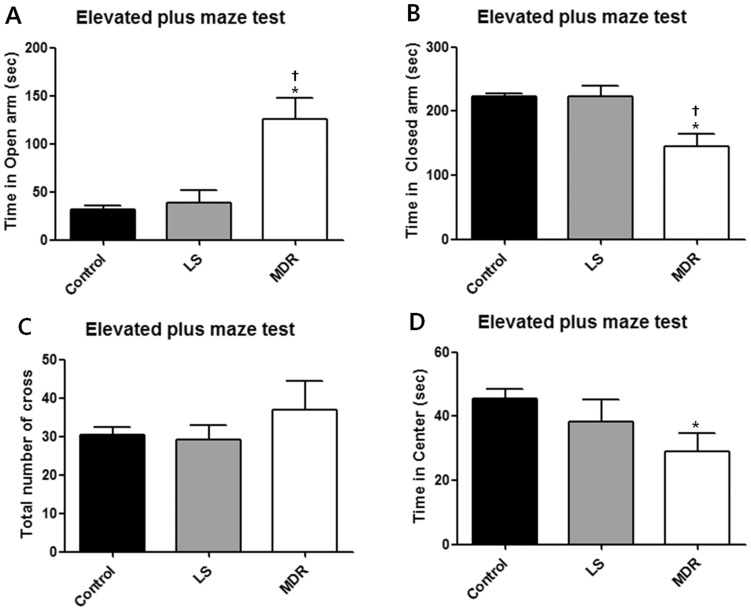
Behavior of mice in each group in the elevated plus maze test. Data are shown as mean ± SEM for the elevated plus maze test (control, n = 10; LS group, n = 12; MDR group, n = 6). (A) illustrates the time that mice spent in the aversive open arms of the maze during 5 min of the test. (B) illustrates the time that mice spent in the closed arms of the maze during the test. (C) illustrates the number of crossings observed during the test. (D) illustrates the time that mice spent in the center of the maze during the test. (*) represents a significant individual difference when compared to the normal group (p<0.05, Mann-Whitney U test). (†) represents a significant individual difference when compared to the LS group (p<0.05, Mann-Whitney U test).

In the elevated plus maze test, the MDR group also showed clearly different behavioral characteristics from the LS and control groups. The MDR group spent more time in the open arms (*p*<0.05) (Fig. 3A) and less time in the closed arms (*p*<0.05; Fig. 3B) than the control and LS groups, without any significant difference in crossing numbers between each groups (Fig. 3C). In addition, the MDR group spent less time in the center than the control group (*p*<0.05; Fig. 3D). The LS group showed similar behaviors with the control group in the elevated plus maze.

**Figure 4:**
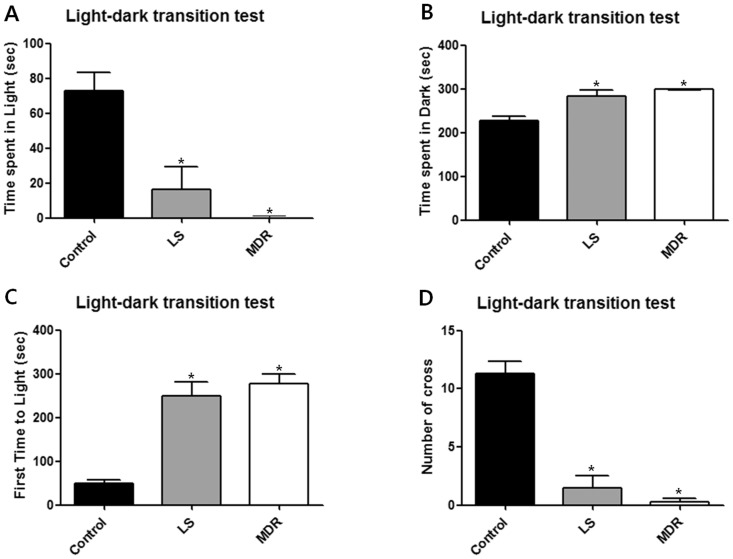
Behavior of mice in each group in the light-dark transition test. Data are shown as mean ± SEM for the light-dark transition test (control, n = 7; LS group, n = 11; MDR group, n = 7). (A) illustrates the time that mice spent in the light compartment during 5 min of the test. (B) illustrates the time that mice spent in the dark compartment during the test. (C) illustrates the time spent prior to the first entry to the light compartment. (D) illustrates the number of crossings between the light and dark compartments during the test. (*) represents a significant individual difference when compared to the normal group (p<0.05, Mann-Whitney U test).

In the light-dark transition test, both the LS and MDR groups spent less time in the light compartment (*p*<0.05; Fig. 4A) and more time in the dark compartment (*p*<0.05; Fig. 4B) compared to the control group. Latency to enter the light compartment was also significantly increased (*p*<0.05; Fig. 4C) and the number of crossings was lower (*p*<0.05; Fig. 4D) in both the LS and MDR groups than in the control group. There was no significant difference between the LS and MDR groups.

### Different miRNA expression profiles according to the responsiveness to antiepileptic drugs

**Figure 5:**
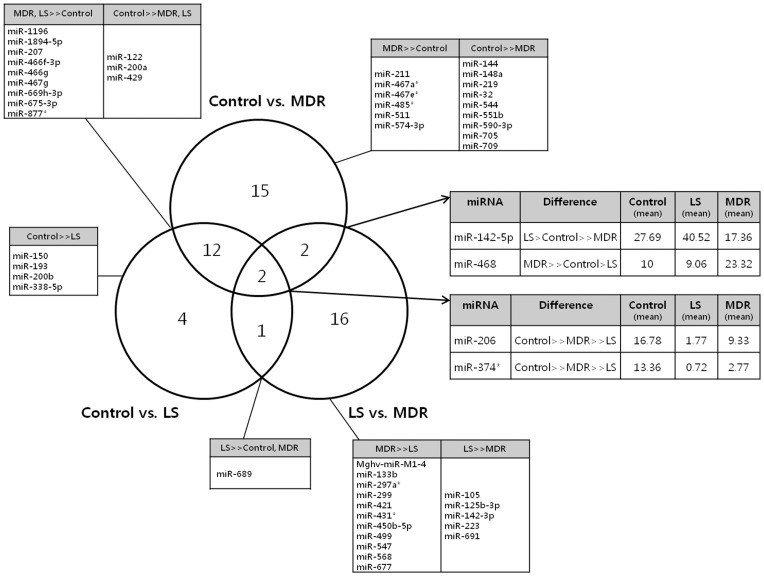
Venn diagram describing miRNA expression patterns in each group. The Venn diagram represents the number of differently expressed miRNAs observed in the comparisons among the three groups (control, LS, and MDR groups). The tables give the specific miRNAs that showed significantly different expression levels among groups. (>>) represents a significant intergroup difference of more than twofold changes and a P-value <0.05. (>) represents non-significant intergroup differences in miRNA expression levels.

RNA samples from 11 animals, 3 from the control group and 4 from each LS and MDR groups were analyzed. The Venn diagram (Fig. 5) summarizes the differently expressed miRNAs among the three groups. Expression levels of 31 miRNAs were different between control and MDR group, and 21 miRNAs were different between the LS and MDR groups. We focused on the 4 miRNAs (miR-206, miR-374, miR-142-5p, and miR-468) which were different between the control and MDR groups, as well as between the LS and MDR groups. We also identified 33 miRNAs that were differently modulated between the control and MDR groups, and between the LS and MDR groups, but not between the control and LS groups, on the assumption that these changes might reflect characteristics of the DRE.

## Discussion

We demonstrated a subgroup of mice resistant to both LEV and VPA in the pilocarpine-induced epilepsy model. This subgroup showed distinctive behavioral characteristics and different miRNA expression patterns as compared to AED responsive mice.

Some of the epileptic animals demonstrated significantly different responsiveness to LEV and/or VPA administration, although we used the same procedure in the syngenic mice to induce epilepsy. Because we administered AEDs to every epileptic mouse without any preceding selection procedure, the LS and MDR groups were naturally selected according to the individual responsiveness to LEV and VPA. Moreover, the animals tended to be either very responsive or extremely unresponsive to each AED. This trend has been described in a previous study [10], but the exact mechanism is still unknown. We additionally noticed that when a mouse is resistant to a specific AED, seizures tended to increase during the administration of that AED. This tendency was consistently observed in both LRVS and MDR groups, and during both LEV and VPA administrations. After all, the most notable observation in this study was discovering that the pharmacoresistant mice can be naturally selected by simply administering two AEDs in sequence in the well-known pilocarpine-induced epilepsy model.

Among the most widely used post-SE models of TLE, there have been some reports about specific subgroups that were nonresponsive to single AED treatments [9], [10], [12], and one report showed that resistance to phenobarbital extended to phenytoin [13]. To our knowledge, this study is the first to demonstrate a subgroup that was resistant to sequential administration of 2 AEDs in a chemically-induced post-SE model. We propose a subgroup of pilocarpine model resistant to both LEV and VPA as a novel model of DRE that fulfills the precise definition of pharmacoresistance.

The target hypothesis and drug transporter hypothesis are the two main hypotheses explaining the mechanism for AED resistance [2]. The target hypothesis postulates that the molecular alteration of the AED target leads to pharmacoresistance [30], while the drug transporter hypothesis states that pharmacoresistance is caused by overexpression of a multidrug transporter (P-glycoprotein, ABCB1) that prevents AEDs from entering the brain in sufficient concentrations. Considering the diverse targets of the various AEDs, resistance to multiple AEDs are usually explained by the drug transporter hypothesis [12].

LEV has a unique mechanism of action targeting synaptic vesicle protein SV2A [31]. VPA possess multiple mechanism of action and acts on GABAergic system, T-type calcium channel, voltage-gated sodium channels, NMDA type glutamate receptors and so on [32], [33]. Although multiple target alterations are not unlikely in epileptic tissue and respective alterations may have led to resistance to multiple AEDs, we regarded it was insufficient to explain our experimental findings by the target hypothesis. Drug transporter hypothesis is also implausible because neither drug is a substrate for P-glycoprotein and other multidrug transporters [34], [35].

A recently proposed hypothesis of pharmacoresistance in epilepsy is the intrinsic severity hypothesis [36]. The main concept is that a higher seizure frequency in the early phase of epilepsy increases the risk of drug resistance. However, the intrinsic severity hypothesis also has weak points. There are patients who start with only few seizures and become pharmacoresistant [37]. Also in our animal model experience [24], the initial severity of the seizure did not correlate with future drug response (data not shown). Therefore, we assumed that another mechanism underlying the pharmacoresistance were present.

Psychiatric disorders frequently occur in patients with epilepsy, and behavioral alterations have been reported in epileptic animals [18], [38]–[40]. It is known that the epileptic mice of pilocarpine model exhibit increased level of anxiety in various anxiety tests, except the elevated plus maze test [18], [38]. We performed four widely used behavioral tests to compare the behavioral nature of AED responsive and resistant mice. In the open field test, the AED responsive LS group and AED resistant MDR group both spent less time in the aversive center field than the control mice. In the light-dark transition test, the LS and MDR groups both spent more time in the dark compartment and less time in the light compartment, their first time to the light compartment was extended, and their number of crossings was decreased when compared to the control group. These findings indicate that the epileptic mice were more anxious than the controls, in concordance with the aforementioned previous studies [18], [38].

Interestingly, the MDR group demonstrated considerably different behaviors in the open-field, object exploration, and elevated plus maze tests. During the open-field and object exploration tests, the MDR group alone showed increased locomotor or exploratory activity compared to the control or LS groups. Additionally, in the elevated plus maze test the MDR group spent much more time in the open arms and less time in the center compared to the control and LS groups. We regarded these findings as behavioral features distinguishing the MDR and LS groups.

The elevated plus maze is a well-known and widely used model for assessing anxiety-related behavior and locomotor activity in rodents [41]. However, this test not only assess factors related to anxiety and locomotor activity, but also evaluates the subjects' risk assessment and decision making by measuring the time spent in the center [42]. Significantly decreased time spent in the center together with increased time spent in the open arms in the MDR group implies impairment of their risk assessment and decision-making ability, rather than anxiety-like behavior.

The increased locomotor activity of MDR group may have influenced the result of behavioral tests. However, while the locomotor activity of MDR group was increased in the open field test, the number of crossing in the elevated plus maze test did not differ from other groups. Moreover in light-dark transition test, number of crossing was decreased in MDR group, so we did not consider this phenomenon as a putative bias in the behavioral tests.

Considering that behavioral alteration is unlikely to occur in response to AED treatment, and on the basis of a recent research [23], we concluded that the behavioral alteration started in relation to pilocarpine-induced SE. The miRNA expression profile is altered after pilocarpine-induced SE [43], and the behavior of a mouse can be affected by epigenetic mechanisms [44]. Therefore, we hypothesized that the behavioral alterations observed in each epileptic group were the outcomes of changed miRNA modulation after pilocarpine-induced SE. Through the miRNA microarrays in each group, we discovered 4 microRNAs (miR-206, miR-374, miR-142-5p, and miR-468) that were significantly altered in the MDR group when compared to both the LS and control groups. Although experimental evidence is lacking in epilepsy, miRNAs are known to be related to the pathogenesis of drug resistance in cancer [45]. It is believed that similar mechanism also plays a role in DRE [46]. Moreover, considering the fact that syngenic mice showed different drug responsiveness and altered behavior after pilocarpine-induced SE supports the presence of epigenetic mechanism. We can infer that the modulation of abovementioned 4 miRNAs may play a key role in developing DRE, as well as in the behavioral alterations.

We have reported that miR-206 reduces brain-derived neurotrophic factor (BDNF) protein levels in the brain [47]. BDNF expression is increased after seizure, and BDNF plays an important role in epileptogenesis by increasing neuronal excitability [48]. It is understandable that the epileptic mice showed reduced miR-206 expression in our study, considering its effect on BDNF. However, simply regarding DRE as a more severe form of epilepsy, the finding that miR-206 expression was more decreased in AED responsive mice should be explained in the future.

miR-374 also showed an altered expression pattern similar to miR-206's, with epileptic mice showing decreased expression and the most differences in the AED responsive mice. The altered patterns of miR-468 and miR-142-5p are interesting because AED responsive and resistant mice demonstrated opposite responses. We hypothesize that miR-468 and miR-142-5p contribute to a unique feature of pharmacoresistance. However, the functions of miR-374, miR-468, and miR-142-5p are not clearly known. A full mRNA expression analysis should be performed, and the detailed functions of these miRNAs and their impact on the behavioral alterations should be elucidated in the future. Moreover, understanding the roles of the 33 miRNAs that were significantly changed in the MDR group, but not between the control and LS group would help us understand the pathogenesis of DRE. Once the target miRNA is revealed, therapeutic applications modulating the expression of the specific miRNA could be attempted, the potential for which our group has shown elsewhere in a neurodegenerative disorder [47].

In conclusion, we have generated a novel model of DRE. In the pilocarpine-induced epilepsy model, when LEV and VPA are administered serially, a naturally selected DRE mouse is produced, which exhibits unique behavioral features. The mechanism of this pharmacoresistance can be explained by none of the target hypothesis, drug transporter hypothesis or intrinsic severity hypothesis. Our data suggest the epigenetic mechanism for developing pharmacoresistance, but a detailed explanation needs to be elucidated in the near future.
